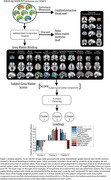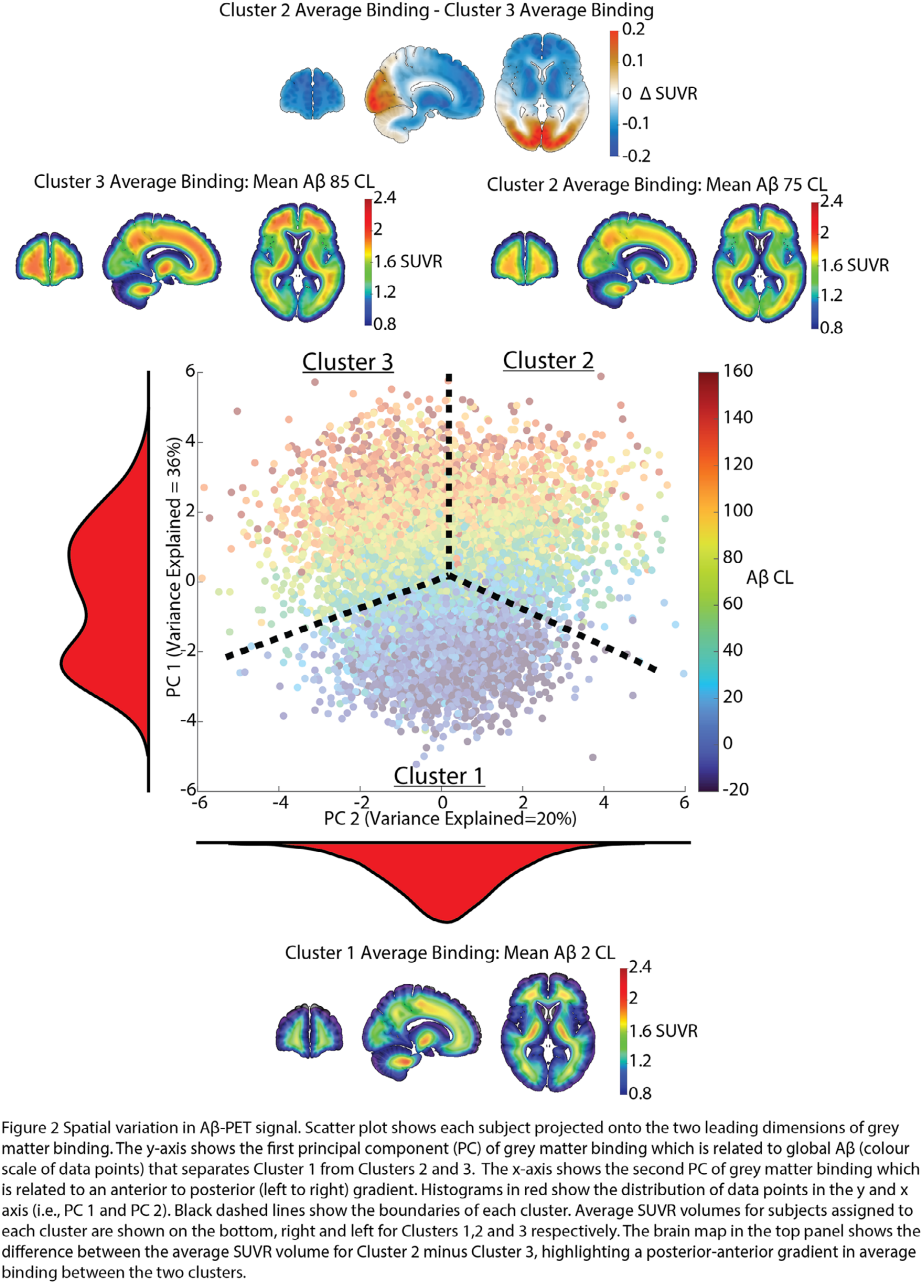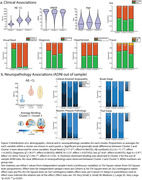# Data‐driven analysis of 10,361 amyloid‐PET scans from the IDEAS study reveals two primary axes of variation

**DOI:** 10.1002/alz.091027

**Published:** 2025-01-09

**Authors:** Joseph Giorgio, Nidhi S Mundada, Ganna Blazhenets, Jhony Alejandro Mejía‐Perez, Daniel R. Schonhaut, Maria C. Carrillo, Lucy Hanna, Constantine Gatsonis, Andrew March, Charles Apgar, Barry A. Siegel, Bruce E Hillner, Rachel A. Whitmer, William J. Jagust, Gil D. Rabinovici, Renaud La Joie

**Affiliations:** ^1^ University of California, Berkeley, Berkeley, CA USA; ^2^ The University of Newcastle, Callaghan, NSW Australia; ^3^ Memory and Aging Center, Weill Institute for Neurosciences, University of California, San Francisco, San Francisco, CA USA; ^4^ University of California, San Francisco, San Francisco, CA USA; ^5^ Alzheimer's Association, Chicago, IL USA; ^6^ Brown University, Providence, RI USA; ^7^ American College of Radiology, Reston, VA USA; ^8^ Mallinckrodt Institute of Radiology, Washington University School of Medicine, St. Louis, MO USA; ^9^ Virginia Commonwealth University, Richmond, VA USA; ^10^ University of California, Davis School of Medicine, Sacramento, CA USA; ^11^ Lawrence Berkeley National Laboratory, Berkeley, CA USA; ^12^ Weill Institute for Neurosciences, University of California, San Francisco, San Francisco, CA USA

## Abstract

**Background:**

The variability in the regional distribution of Aβ‐PET signal and its relation to clinical features is debated. We used data‐driven approaches to uncover heterogeneity in cortical Aβ‐PET signal from a large representative sample collected through the IDEAS study.

**Methods:**

We analysed cross‐sectional Aβ‐PET collected from 10,361 patients with MCI or mild dementia scanned in 295 PET facilities using one of the 3 FDA‐approved tracers. Central image processing resulted in template‐space SUVR images (reference: whole cerebellum) and centiloid (CL) values. Spatial independent component analysis was used to decompose SUVR volumes into 40 independent components. After excluding noise components, participants’ scores were extracted for each of the remaining 11 grey matter (GM) components describing cortical and subcortical binding. K‐means clustering was used on these GM component scores to assign each participant to different Aβ‐PET clusters based on GM binding (Figure 1).

**Results:**

Three informative clusters of PET binding were estimated. Cluster 1: Aβ‐(n=4729, CL mean=2±23) with low GM binding, and two Aβ+ clusters; Cluster 2(n=2484, CL mean=76±34) and Cluster 3(n=3148, CL mean=86±32). Subtracting average SUVR of Clusters 2 and 3 showed they differed along a posterior‐anterior gradient with Cluster 2 showing an occipital predominant pattern. Principal component analysis conducted on the GM scores confirmed two dominant axes of variation separated the clusters, a Aβ‐ to Aβ+ axis and, an anterior‐posterior axis (Figure 2). Statistically significant but weak differences were observed between the two Aβ+ Clusters (2 vs. 3); Visual Read (positive: 95% vs. 92%); Clinical Stage (dementia: 47% vs. 41%); Age (76.9±6.4 vs. 75.9±6.2), however, most clinical variables showed no differences (Figure 3a). 48 ADNI participants with Aβ‐PET and post‐mortem neuropathology data (11 Female, Age mean=79.7±7.4, PET‐Death mean=2.3±1.7years; Aβ‐CL mean=71.2±55.5; APOE4(0/1/2)=22/21/5; Diagnosis(CN/MCI/AD)=6/8/32) were applied to the model fit on IDEAS data. Qualitatively, no differences in neuropathology were observed between the two Aβ+ Clusters (Figure 3b).

**Conclusion:**

Data driven classification of Aβ‐PET reveals two primary axes reflecting Aβ load and anterior‐posterior binding, with the later not clearly related to clinical or pathological variation. Future work will apply new data to this model and investigate if this spatial variation in Aβ‐PET is related to longitudinal changes in pathology.